# Epidemiology of Hepatitis C Virus Infection and Risk Factor Analysis in the Hebei Province, China

**DOI:** 10.1371/journal.pone.0075586

**Published:** 2013-09-19

**Authors:** Yuliang Zhao, Liping Shen, Jingchen Ma, Zhao Gao, Xu Han, Shunxiang Qi, Qi Li

**Affiliations:** 1 Center for Disease Control and Prevention of Hebei Province, Shijiazhuang, China; 2 National Institute for Viral Disease Control and Prevention, Chinese Centre for Disease Control and Prevention, Beijing, China; Duke University, United States of America

## Abstract

**Background:**

In 1985, a hepatitis C virus (HCV) outbreak caused by plasmapheresis donation was reported in the Hebei Province, China. However, studies assessing the epidemic features and risk factors of HCV in the general population of Hebei have been limited until now.

**Methods:**

The multicenter cluster sampling method was used to collect samples. The participants were interviewed. Relevant information was obtained from the general population using a standardized questionnaire, and association and logistic regression analyses were conducted. Serum samples were taken to test anti-HCV by enzyme immunoassays.

**Results:**

A total of 4562 participants from 11 cities of the Hebei Province were enrolled. The average anti-HCV positive rate was 0.62% (29/4562), which was 1.07% in the rural population, compared with 0.22% in the urban population. The anti-HCV positive rate in the 40–59-year age group was higher than in those aged <40 years. History of blood transfusion and transmission in families were the main risk factors for HCV infection in this area.

**Conclusion:**

The anti-HCV positive rate in Hebei has decreased significantly from that two decades ago. Safety of blood products and health education about HCV still need to be improved.

## Introduction

Hepatitis C virus (HCV) infection is a major cause of chronic hepatitis, cirrhosis and hepatocellular carcinoma [1,2]. Currently, 130–170 million people worldwide are infected with HCV, and the annual increase is approximately 3.5 million [3]. According to a multicenter epidemiological study in China between 1991 and 1995, the average HCV prevalence in the general population was 2.2% (range: 0.52–3.15%), amounting to approximately 26.4 million infected individuals [4]. Another representative survey of HCV infection in China was carried out in 1992. In that survey it was found that the average anti-HCV positive rate in China was 3.2%, with 2.26% in the Hebei Province [5,6]. HCV transmission is associated with blood transfusion, sexual contact, and injecting drug use. In the Hebei Province, HCV infection was first reported in 1985 in a serious outbreak incident caused by plasmapheresis donation [7,8]. Recently, blood products have been controlled strictly in China, and a similar event has not been reported in the Hebei Province. Epidemiology surveys of HCV infection are important for prevention and treatment of the disease. However, little is still known about HCV infection among the general population in the Hebei Province. More attention needs to be paid to the study of risk factors for HCV infection in this area. In our study, to assess the epidemic features of HCV prevalence and risk factors for infection, we conducted an survey among the general population in the Hebei Province.

## Material and Methods

### Sampling method

The total population in the Hebei Province is nearly 72,000,000 and 56% of the people comprise the rural population. There are 11 administrative cities in the Hebei Province. The proportional to population size cluster sampling method was used in this study. One county representing the rural population and one district representing the urban population were selected in every city. A total of 11 counties and 11 districts were enrolled. According to sample size formula and loss to follow-up, about 5000 subjects should be enrolled in our study. The sample size of every county and district was no less than 220.

### Data collection

In August 2010 to May 2011, trained social workers conducted a house to house interview using a complete and detailed questionnaire to collect information about the study subjects and assess potential risk factors for HCV infection. The questionnaire included questions such as educational level, history of liver disease, family medical history, blood donation/transfusion history, past use of glass syringes, and surgical intervention, et al.

The survey protocol conformed to the ethical guidelines of the 1975 Declaration of Helsinki and was approved by the Hebei CDC Ethics Committee, and all study work was performed in accordance with national ethic regulations. Study participants were informed of the study purpose and of their right to keep information confidential. All participants provide their written consent to participate in this study.

### Detection of anti-HCV antibodies

A 5-ml serum sample from each person was collected, separated, labeled, and stored frozen at -20°C within 4 h of collection. All the samples were screened for anti-HCV antibodies using commercial third-generation enzyme immunoassays (ELISA) from two manufacturers (Wantai Core Anti-HCV ELISA kit, C20101120, Beijing, China; Xinchuang Core Anti-HCV ELISA kit, C201095819, Xiamen, China). Sera that reacted with both of the commercial kits were considered to be positive, and positive findings were considered to be an indicator of previous HCV contact.

### Statistical analysis

Statistical tests were performed using EPI Info 3.51 software. The χ^2^ test or Fisher’s exact test was used to determine whether associations were statistically significant. Risk factors for HCV infection were analyzed by multivariate logistic regression. The crude odds ratio (OR) and 95% confidence interval (95% CI) were calculated to estimate if there were differences in potential risk factors between anti-HCV positive and anti-HCV negative individuals. Comparison between groups should involve multiple comparisons of multiple sample means. A two-sided P value <0.05 was considered to be statistically significant. The anti-HCV positive rate of areas and gender were adjusted according to the data of population census in 2010, which of the whole population was adjusted by ratio of urban and rural populations of the Hebei Province in 2010, which was 1:1.28.

## Results

### General features of participants

A total of 4562 people were enrolled from 11 counties and 11 districts in the Hebei Province, of whom, 2308 (50.61%) were from the urban population, and 2254 (49.39%) from the rural population. There were 2208 (48.39%) male and 2354 (51.61%) female subjects. The age of the participants ranged from 1 to 59 years, with a mean of 33.0 ±16.75.

### Area, age and sexual distribution of anti-HCV positive rate

Twenty-nine individuals were anti-HCV positive. The adjusted anti-HCV positive rate of the whole population was 0.62% (95%CI: 0.39^~^0.85%). The rate in the rural population was 1.07% (95% CI: 0.53^~^1.33%), which was higher than that in the urban population (0.22%; 95% CI:0.03^~^0.42%). The difference was significant (χ^2^=12.98, *P*<0.001). The adjusted anti-HCV positive rate for male subjects was 0.38% (95% CI: 0.12–0.64%), compared with 0.75% (95%CI: 0.40–1.10%) in female subjects. There were no significant differences between the sexes (χ^2^=2.26, *P*>0.05).

### Risk factors for HCV infection

Single factor analysis was carried out for 14 possible risk factors such as age, dental care, blood donation, sharing syringes, and family members who are anti-HCV positive. Among the 29 anti-HCV-positive individuals, 10 had a history of blood transfusion and using other blood products. These six risk factors were related to HCV infection (Table 1). Further analysis by multivariate analysis showed that five of the risk factors were significantly related to HCV infection, Further analysis by multivariate analysis showed that five of the risk factors were significantly related to HCV infection, which including family members are anti-HCV positive(OR=13.74, 95%CI 4.92^~^38.35), blood transfusion(OR=9.473, 95%CI 3.32^~^27.02), live in rural area (OR=4.72, 95%CI 1.75^~^12.70), blood donation (OR=3.33, 95%CI 1.10^~^10.06) and age is 40-59 years old (OR=2.69, 95%CI 1.19^~^6.08).

**Table 1:** Single factor analysis of HCV infection and related risk factors.

**Risk factor**	**No. of samples**	**No. of positive**	**Positive rate**	**OR(95%CI**)	**P value**
**Area**					
Rural	2254	24	1.07	4.96(1.97^~^14.82)	<0.001
Urban	2308	5	0.22		
**Age group(year**)					
40^~^59	1881	20	1.06	3.19(1.38^~^7.57)	<0.01
1^~^39	2681	9	0.34		
**Blood Transfusion**					
Yes	116	6	5.17	10.42(3.72^~^27.64)	<0.001
No	4417	23	0.52		
**Using other blood product**					
Yes	77	4	5.19	9.65(2.77^~^30.17)	<0.01
No	4426	25	0.56		
**Blood donation**					
Yes	246	5	2.03	3.7(1.23^~^10.33)	<0.05
No	4307	24	0.56		
**Family members are anti-HCV positive**					
Yes	70	6	8.57	18.22(6.41^~^49.21)	<0.001
No	4492	23	0.51		

## Discussion

HCV infection represents a worldwide healthcare problem. HCV infection shows clear differences in prevalence among geographic regions and individuals of different ages, and according to World Health Organization data [9]. Last representative survey of HCV infection was carried out in 1992. In order to know recent HCV infection status in the Hebei Province, a epidemiological survey was carried out in 2010.

The results of the survey showed that the anti-HCV-positive rate in the general population in Hebei was 0.62%, which was lower than the previously reported rate of 2.26% in 1992 [5,6]. This dramatic difference may have been due to improvements in medication and quality of life in recent years, especially the safety of blood transfusion. It could also be that there were a large number of false-positive assay results in 1992 because the quality of local anti-HCV ELISA kits was not stable at that time.

There were significant regional differences in the anti-HCV positive rate in Hebei Province in our study. We found a higher rate in the rural population than in the urban population. The anti-HCV positive rate differed significantly between age groups, with a higher rate in those aged 40–59 years compared with those aged <40 years. The increase in anti-HCV positive rate with age was consistent with previous studies [10,11]. Young people have fewer opportunities to be infected with HCV. Also, there are now fewer people who can transmit HCV infection than before because of control and prevention strategies.

The main risk factors for HCV infection were family members who are anti-HCV positive, blood transfusion, blood donation, and living in a rural area. The fact that blood transfusion was still an important risk factor reminds us that the safety of blood products still needs to be improved. In China, several case–control studies have been performed to compare HCV-positive and HCV-negative individuals, and it has also been found that the most prevalent risk factor in the HCV-positive general population was a history of blood transfusion [12,13]. Transmission in families was another important risk factor, which emphasizes the need to improve health education. With the implementation of mandatory HCV screening of blood and blood products in the early 1990s, the number of post-transfusion infections has decreased dramatically, while intravenous drug use has accelerated in China [14,15]. The result indicated the need for extensive studies to determine the epidemiology of HCV infection and to develop appropriate prevention programs to control transmission of the virus.

Detailed and careful interview that carried out by house to house is the strength of this survey. However, relatively small sample size is the limitation. In the following study we will expand the sample size and make further analysis.

HCV infection remains a serious public health problem in China. Strengthening administrative regulation of medical practice, especially in rural areas, and providing appropriate education to the public about HCV infection and its transmission should be given higher priority in public health policy.
